# Implementation status and barriers to multimodal management of low back pain among physical therapists: A cross-sectional study

**DOI:** 10.1038/s41598-026-50076-2

**Published:** 2026-04-24

**Authors:** Ryo Miyachi, Takaaki Nishimura, Masahiro Noguchi, Akio Goda, Yuji Kanazawa, Koshi Shimizu, Hisashi Narimiya, Sachiko Madokoro, Kentaro Mori, Takashi Kitagawa, Yayoi Kojima

**Affiliations:** 1 Faculty of Health and Medical Sciences, Hokuriku University, 1-1 Taiyogaoka, Kanazawa, 920-1180 Ishikawa Japan; 2 Well-Being Research Team, Hokuriku University, Kanazawa, Japan; 3 Keyaki Orthopedic Clinic, Kanazawa, Japan; 4 Faculty of Health Sciences, Institute of Medical, Pharmaceutical, and Health Sciences, Kanazawa University, Kanazawa, Japan; 5 Department of Rehabilitation, Ishikawaken Saiseikai Kanazawa Hospital, Kanazawa, Japan; 6 Department of Physical Therapy, School of Health Sciences, Shinshu University, Matsumoto, Japan; 7 Faculty of International Communication, Hokuriku University, Kanazawa, Japan

**Keywords:** Low back pain, Physical therapists, Multimodal management, Questionnaire survey, Implementation barriers, Health care, Health occupations, Medical research, Risk factors

## Abstract

This study aimed to investigate the implementation of multimodal assessments and interventions for low back pain (LBP) among physical therapists and identify the reasons and participants’ background characteristics associated with non-implementation. A cross-sectional online survey was conducted among members of the Ishikawa Physical Therapy Association in Japan with clinical experience in managing patients with LBP. A total of 140 valid responses were analyzed. The questionnaire included multiple-choice items on respondent and patient characteristics, awareness of LBP-related factors (five domains: biomechanical, psychological, social, lifestyle, and metabolic; 18 sub-items), implementation and reasons for non-implementation of assessments and interventions, and types of interventions provided. Comparisons among the five domains showed that awareness and implementation were significantly higher for biomechanical factors than for the other domains, whereas for psychological and metabolic factors, they were significantly lower. The main reasons for non-implementation were a lack of knowledge or skills, limited time, and delegation to other professionals. Discriminant analysis showed that these reasons varied depending on work setting and patient characteristics. These findings suggest that physical therapy for LBP tends to focus on biomechanical factors, whereas psychological, lifestyle, and metabolic perspectives are less frequently integrated into practice. The results also indicate that educational and organizational factors, along with role boundaries and interprofessional considerations, may influence how these domains are addressed in clinical settings.

**Supplementary Information:**

The online version contains supplementary material available at 10.1038/s41598-026-50076-2.

## Introduction

The lifetime prevalence of low back pain (LBP) is approximately 80%, making it a common and socially impactful health problem worldwide^1–3^. LBP is characterized by high recurrence and chronicity rates, and most individuals experiencing activity-limiting LBP subsequently report recurrent episodes^3^. LBP reduces individuals’ quality of life and imposes substantial socioeconomic burden through increased healthcare expenditures and reduced work productivity^1–4^.

The biopsychosocial model, originally proposed by Engel^5^, was later developed and applied to LBP by Waddell^6^. This model conceptualizes pain as arising from the interaction of biological, psychological, and social factors, in contrast to the traditional biomedical model, which primarily focuses on biological mechanisms. This is widely accepted, particularly in the context of LBP research and clinical practice^7^. As previously reported, biomechanical factors, including trunk muscle strength and range of motion^8–12^; psychological factors, including depressive symptoms and fear of movement^13–15^; social factors, including work environment and stress^16,17^; and lifestyle and metabolic factors, including diet, sleep, physical activity, and obesity^18–20^, are associated with LBP onset and persistence. Furthermore, multimodal management approaches addressing multiple contributing factors effectively improve outcomes in individuals with LBP^21–28^.

Although multimodal assessment and intervention strategies for LBP have been widely studied^21–28^, their implementation in clinical practice remains underexplored. In addition to the variability in physical therapists’ knowledge of LBP evidence^29,30^, several factors, including challenges in remaining current, beliefs that evidence may not directly apply to practice, time constraints, and mismatches with patient expectations, have been reported to hinder evidence-based guideline-concordant interventions^31–33^. Notably, most previous studies on the barriers to implementing multimodal LBP management have primarily focused on psychological factors^34,35^. Few studies have systematically examined multiple domains, including biomechanical, social, lifestyle, and metabolic factors, to identify which factors are less likely to be assessed or addressed and why. This knowledge gap is a critical issue from an implementation science perspective, with important implications for designing physical therapy education curricula and optimizing multidisciplinary collaboration models.

Therefore, this study aimed to clarify physical therapists’ awareness and their implementation of assessment and intervention across five major domains–biomechanical, psychological, social, lifestyle, and metabolic factors–in LBP management as well as their reasons for non-implementation. We also examined how these reasons varied with the clinical context, including the characteristics of physical therapists and their patients. Through this investigation, we sought to conceptualize the barriers to implementing multimodal management not as individual physical therapists’ shortcomings but as structural challenges arising from the complex interplay of educational, organizational, and contextual factors.

## Methods

### Study design and participants

The study protocol was not registered. This study employed a cross-sectional observational design, using an online questionnaire administered between February and March 2025. This study was reported in accordance with the STROBE guidelines for cross-sectional studies. The survey targeted 1,184 physical therapists registered with the Ishikawa Physical Therapy Association. This association includes physical therapists affiliated with major acute care hospitals, rehabilitation hospitals, and orthopedic clinics throughout Ishikawa Prefecture, and is considered to represent the main working population of physical therapists who provide clinical care for patients with LBP in this region. In Japan, acute care hospitals typically provide early-stage rehabilitation following injury or surgery; rehabilitation hospitals focus on more intensive and longer-term rehabilitation; while orthopedic clinics mainly deliver outpatient care for musculoskeletal conditions.

Participant recruitment for this study was conducted through two channels: an announcement distributed to all association members via an email newsletter and a request to facility representatives to further disseminate the information within their affiliated institutions. The exclusion criteria were physical therapists who reported rarely treating patients with LBP and those whose native language was not Japanese. No predefined target sample size was calculated for this study. As this was an exploratory cross-sectional survey, all eligible members of the Ishikawa Physical Therapy Association were invited to participate, and the final sample size was determined based on the number of respondents.

### Ethical considerations

Before beginning the survey, the participants were informed on the introductory page of the questionnaire about the study’s purpose and content, the voluntary nature of participation, the exclusive use of collected data for research purposes, and the strict confidentiality with which personal information would be handled. Informed consent was obtained from all participants electronically by having them click the “Agree” button. This study was approved by the Ethics Committee of Hokuriku University (approval no. 2024-31). All procedures were conducted in accordance with the relevant guidelines and regulations, including the ethical principles for research involving human participants.

### Online questionnaire

The online questionnaire was initially developed by the first author according to domestic and international LBP management guidelines alongside key components of multimodal management^8–37^. The candidate items were generated by the first author through a review of clinical guidelines and relevant literature, by identifying factors associated with the onset, persistence, and recurrence of LBP. These items were subsequently organized into five domains representing the key components of multimodal LBP management: biomechanical, psychological, social, lifestyle, and metabolic factors. To ensure content validity, an expert review was conducted by three physical therapists with over 15 years of clinical experience and one expert in psychological survey methodology. The reviewers independently examined each item in terms of clinical relevance and clarity of wording, and the questionnaire was refined based on their feedback. A cognitive pretest was subsequently conducted with six physical therapists to confirm the item comprehensibility. Participants independently completed the draft questionnaire, and no items required major revisions. Final decisions regarding item inclusion and wording were made collectively by the research team after an expert review and a pretest. This questionnaire was independently developed for this study and was not based on any previously published or copyrighted instrument; therefore, no permission or license was required. Because the questionnaire was developed as a pragmatic instrument for this study, formal scale development procedures (e.g., quantitative content validity indices or full psychometric validation) were not conducted.

The final questionnaire consisted of the following six sections (Supplementary Material 1):

1. Respondent characteristics.
2. Patient characteristics.
3. Awareness (knowledge) of LBP-related factors.
4. Implementation of assessment and reasons for non-assessment.
5. Implementation of intervention and reasons for non-intervention.
6. Types of interventions currently provided.

Awareness was defined as whether the respondents recognized each factor as being related to LBP. Participants were presented with a list of factors and asked to select all the items they considered relevant.

LBP-related factors were classified into five domains: biomechanical, psychological, social, lifestyle, and metabolic, comprising 18 subitems. Factors were categorized based on their primary clinical relevance, although some may overlap across domains. Assessment implementation was defined as the routine collection of information through interviews or examinations, whereas intervention implementation was defined as the provision of advice, exercises, education, or behavioral modification support to improve the corresponding factor.

### Statistical analysis

Statistical analyses were performed using SPSS Statistics for Windows, version 28 (IBM Corp., Armonk, NY, USA) and HAD (version 18_008)^38^. HAD is a statistical software package equipped with a graphical user interface that is widely used in education, psychology, and healthcare research.

Descriptive statistics were used to summarize the responses for each item. Differences among the five domains were examined using chi-square tests with Holm correction. Effect sizes were calculated using the φ (phi) coefficient and interpreted as small (0.1), medium (0.3), and large (0.5).

For each domain, the “implementation of assessment or intervention” was defined as a positive response (“implemented”) to at least one subitem within that domain.

To explore the latent structure of respondents’ and patients’ characteristics, quantification theory type III (a variant of multiple correspondence analysis [MCA] suitable for categorical data) was performed, and the first and second dimensions were extracted as interpretive variables^39^. Furthermore, to clarify the relationships of reasons for non-implementation with respondents’ and patients’ background characteristics, discriminant analysis was conducted using the quantification theory type III-derived scores as independent variables, and reasons for non-implementation of LBP assessment and intervention as dependent variables.

The dependent variables were the reasons for non-implementation, including “do not know how,” “lack of skills,” and “delegated to other professionals.” The independent variables were quantified scores derived from MCA, reflecting facility characteristics, whether the physical therapist mainly treated patients with chronic LBP, and whether patients with lower limb neurological symptoms were mainly treated.

Discriminant analysis was only performed when items had at least five responses per independent variable^40^. Independent variables were entered using the forced-entry method, and only statistically significant discriminant functions were reported with their p-values, unstandardized discriminant coefficients, and standardized discriminant coefficients. The level of significance was set at *p* < 0.05. As designed, responses with missing data on key variables were excluded from the analysis; however, no missing data were observed in the dataset.

## Results

### Characteristics of respondents and patients

**Fig. 1:**
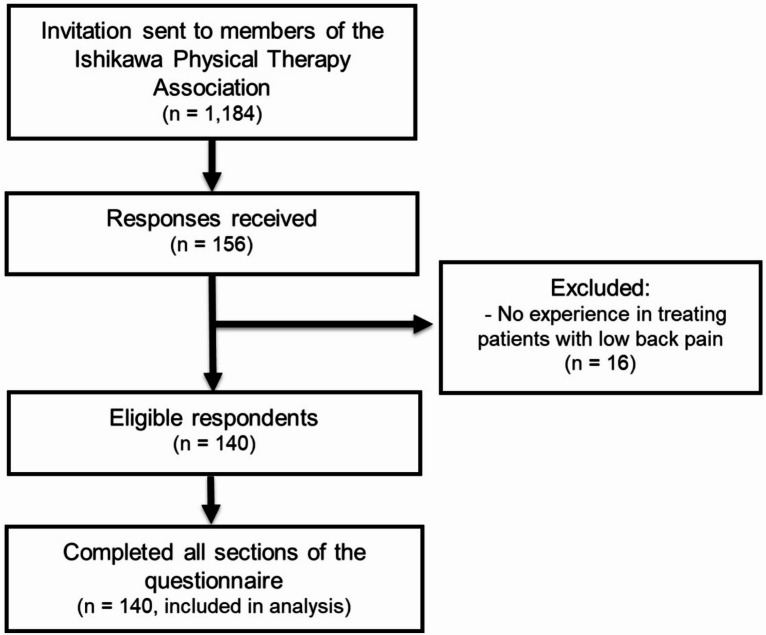
Flowchart of the study participants.

Of 156 physical therapists who responded to the survey, 16 were excluded because they rarely treated patients with LBP; thus, data from 140 physical therapists were included in the final analysis (response rate: 11.8% [*n* = 140/1,184]) (Fig. 1). Most physical therapists were in their 20–30 s, and more than half had less than 20 years of clinical experience. The most common work setting was acute care hospitals (69%), followed by rehabilitation hospitals (49%) (Table 1).

**Table 1:** Characteristics of respondents and the patients they primarily manage.

Characteristics	Category	*n* (%)
Sex	Male	92 (66)
	Female	48 (34)
Age of respondents (years)	20–29	53 (38)
	30–39	51 (36)
	40–49	27 (19)
	50–59	9 (6)
	≥ 60	0 (0)
Clinical experience (years)	0–4	33 (24)
	5–10	30 (21)
	10–14	30 (21)
	15–19	25 (18)
	20–24	13 (9)
	25–29	4 (3)
	30–34	5 (4)
	≥ 35	0 (0)
Work setting ^1^	Acute care hospital	96 (69)
	Rehabilitation hospital	69 (49)
	Orthopedic clinic	19 (14)
	Home-visit rehabilitation service	42 (30)
	Nursing facility	14 (10)
	Sports team	4 (3)
	Others	1 (1)
Frequency of treating patients with low back pain	Almost every day	64 (46)
	Several times a week	43 (31)
	Several times a month	23 (16)
	Several times in 6 months	6 (4)
	Several times a year	4 (3)
Age group of patients with low back pain ^1^	0–19	17 (12)
	20–29	17 (12)
	30–39	18 (13)
	40–49	26 (19)
	50–59	42 (30)
	60–69	63 (45)
	70–79	116 (83)
	≥ 80	123 (88)
Clinical stage of low back pain and leg symptoms^1, 2^	Acute, no leg symptoms	82 (59)
	Chronic, no leg symptoms	103 (74)
	Acute, with leg symptoms	59 (42)
	Chronic, with leg symptoms	84 (60)
Patient condition mainly managed ^1^	Postoperative (inpatient)	55 (39)
	Postoperative (outpatient)	28 (20)
	Non-surgical (conservative) management (inpatient)	95 (68)
	Non-surgical (conservative) management (outpatient)	54 (39)

^1^ Multiple answers were allowed.

^2^ Acute low back pain was defined as symptom duration < 3 months, and chronic low back pain as ≥ 3 months.

The percentages were calculated based on the total number of respondents (*n* = 140).

### Awareness of LBP–related factors

**Fig. 2:**
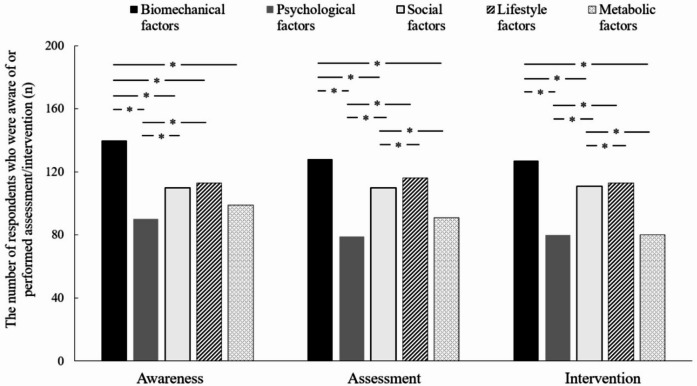
Comparison of awareness, assessment, and intervention rates across five domains (biomechanical, psychological, social, lifestyle, and metabolic factors). * Significant difference between domains (*p* < 0.05).

The number of physical therapists who were aware of each LBP-related factor is presented in Table 2, and differences in the number of physical therapists who were aware of these factors across domains are shown in Fig. 2. Comparisons among domains indicated that awareness of biomechanical factors was significantly higher than that of all other domains (Holm-corrected *p* < 0.01, φ = 0.33–0.47). In contrast, awareness of psychological factors was significantly lower than that of biomechanical factors, social (Holm-corrected *p* = 0.04, φ = 0.16), and lifestyle factors (Holm-corrected *p* = 0.01, φ = 0.18). In the biomechanical domain, high awareness was observed for decreased trunk muscle strength/endurance (98%), overactivation or imbalance of trunk muscles (80%), decreased flexibility of adjacent regions (hip and thoracic spine) (90%), and postural/movement dysfunction (90%). In contrast, relatively low awareness rates were recorded for impaired lumbar motor control (68%) and altered body perception (41%). Regarding psychological factors, awareness of anxiety or depressive symptoms (57%), kinesiophobia (fear of movement) (37%), and low self-efficacy (41%) were relatively low. Among the social factors, work environment (including prolonged desk work and heavy lifting) had the highest awareness rate (77%). In the lifestyle domain, low physical activity (lack of exercise habits) (78%) was the most frequently identified factor, whereas all other items showed awareness rates below 30%. In the metabolic domain, obesity (71%) showed a high awareness rate, while high blood glucose levels (14%) showed a relatively low awareness rate (Table 2).

**Table 2:** Awareness of factors related to low back pain among physical therapists.

Domain	LBP-related factor	*n* (%)
Biomechanical factors	Decreased trunk muscle strength/endurance	137 (98)
	Overactivation or imbalance of trunk muscles	112 (80)
	Decreased flexibility of adjacent regions (hip, thoracic spine)	126 (90)
	Impaired lumbar motor control	95 (68)
	Altered body perception	57 (41)
	Postural/movement dysfunction	126 (90)
Psychological factors	Anxiety or depressive symptoms	80 (57)
	Kinesiophobia (fear of movement)	52 (37)
	Low self-efficacy	58 (41)
Social factors	Stress (family or workplace)	70 (50)
	Work environment (including prolonged desk work, heavy lifting)	108 (77)
Lifestyle factors	Poor sleep (quantity/quality)	48 (34)
	Low physical activity (lack of exercise habits)	109 (78)
	Poor dietary habits	36 (26)
	Frequent alcohol consumption	22 (16)
	Smoking	31 (22)
Metabolic factors	Obesity	99 (71)
	High blood glucose	19 (14)
None of the above		0

Values represent the number (percentage) of respondents who recognized each factor as related to low back pain.

The percentages were calculated based on the total number of respondents (*n* = 140).

Multiple responses were allowed.

LBP: low back pain.

### Assessment of LBP–related factors and reasons for non-assessment

The number of physical therapists who did not assess each LBP-related factor and their reasons are presented in Table 3. Differences in assessment implementation across the factors are shown in Fig. 2. The number of physical therapists implementing assessments of biomechanical factors was significantly higher than that of psychological, social, and metabolic factors (Holm-corrected *p* < 0.05, φ = 0.18–0.40). The psychological and metabolic factors also had significantly fewer physical therapists implementing assessments than biomechanical, social (Holm-corrected *p* < 0.05, φ = 0.15–0.24), and lifestyle factors (Holm-corrected *p* < 0.01, φ = 0.20–0.29).

**Table 3:** Reasons for not assessing low back pain–related factors.

Domain	Factor not assessed	Overall (no assessment)	Do not know how to assess	Lack of assessment skills	Lack of time	Patient discomfort with the assessment	Lack of necessary equipment	Not allowed by facility policy	No eligible patients	Not considered necessary	Delegated to other professionals
**Biomechanical factors**	Decreased trunk muscle strength/endurance	24 (17)	1 (1)	12 (9)	4 (3)	4 (3)	0 (0)	1 (1)	2 (1)	1 (1)	0 (0)
	Overactivation or imbalance of trunk muscles	49 (35)	18 (13)	26 (19)	5 (4)	1 (1)	0 (0)	1 (1)	1 (1)	1 (1)	0 (0)
	Decreased flexibility of adjacent regions (hip, thoracic spine)	27 (19)	9 (6)	15 (11)	3 (2)	2 (1)	0 (0)	1 (1)	0 (0)	0 (0)	0 (0)
	Impaired lumbar motor control	55 (39)	22 (16)	27 (19)	6 (4)	1 (1)	1 (1)	1 (1)	2 (1)	1 (1)	0 (0)
	Altered body perception	66 (47)	23 (16)	30 (21)	6 (4)	3 (2)	3 (2)	1 (1)	2 (1)	5 (4)	1 (1)
	Postural/movement dysfunction	21 (15)	4 (3)	13 (9)	2 (1)	2 (1)	1 (1)	1 (1)	0 (0)	0 (0)	0 (0)
**Psychological factors**	Anxiety or depressive symptoms	87 (62)	23 (16)	28 (20)	18 (13)	6 (4)	0 (0)	1 (1)	3 (2)	6 (4)	17 (12)
	Kinesiophobia (fear of movement)	76 (54)	29 (21)	28 (20)	15 (11)	5 (4)	1 (1)	1 (1)	3 (2)	2 (1)	7 (5)
	Low self-efficacy	88 (63)	40 (29)	30 (21)	14 (10)	5 (4)	1 (1)	1 (1)	0 (0)	2 (1)	7 (5)
**Social factors**	Psychological stress (family or workplace)	71 (51)	24 (17)	23 (16)	7 (5)	6 (4)	1 (1)	1 (1)	6 (4)	8 (6)	11 (8)
	Work environment (including prolonged desk work, heavy lifting)	31 (22)	5 (4)	14 (10)	4 (3)	1 (1)	1 (1)	1 (1)	7 (5)	3 (2)	5 (4)
**Lifestyle factors**	Poor sleep (quantity/quality)	68 (49)	27 (19)	19 (14)	5 (4)	2 (1)	1 (1)	1 (1)	2 (1)	8 (6)	10 (7)
	Low physical activity (lack of exercise habits)	29 (21)	7 (5)	13 (9)	5 (4)	1 (1)	2 (1)	1 (1)	0 (0)	2 (1)	1 (1)
	Poor dietary habits	77 (55)	24 (17)	13 (9)	7 (5)	1 (1)	0 (0)	1 (1)	1 (1)	9 (6)	29 (21)
	Frequent alcohol consumption	79 (56)	22 (16)	13 (9)	2 (1)	2 (1)	0 (0)	1 (1)	1 (1)	16 (11)	29 (21)
	Smoking	74 (53)	19 (14)	11 (8)	4 (3)	2 (1)	0 (0)	1 (1)	1 (1)	16 (11)	26 (19)
**Metabolic factors**	Obesity	50 (36)	11 (8)	17 (12)	3 (2)	2 (1)	2 (1)	1 (1)	1 (1)	2 (1)	18 (13)
	High blood glucose	83 (59)	18 (13)	13 (9)	4 (3)	3 (2)	0 (0)	1 (1)	1 (1)	13 (9)	35 (25)
**All items assessed**		4 (3)	–	–	–	–	–	–	–	–	–

Values represent the number (percentage) of respondents who selected each reason for not assessing each factor.

Percentages were calculated based on the total number of respondents (*n* = 140).

Multiple responses were allowed; therefore, the sum of reasons does not equal the total number of respondents who did not assess each factor.

Among the biomechanical factors, high non-assessment rates were observed for overactivation or imbalance of trunk muscles (35%), impaired lumbar motor control (39%), and altered body perception (47%). The main reasons were “do not know how to assess” (13–16%) and “lack of assessment skills” (19–21%). Regarding the psychological factors, all items exhibited high non-assessment rates, with the main reasons being “do not know how to assess” (16–29%), “lack of assessment skills” (20–21%), and “lack of time” (10–13%). In addition, for anxiety or depressive symptoms, “delegated to other professionals” (12%) was also reported. Among the social factors, stress (family or workplace) showed a high non-assessment rate (51%), primarily due to “do not know how to assess” (17%) and “lack of assessment skills” (16%). For the lifestyle factors, high non-assessment rates were observed for poor sleep (quantity/quality) (49%), poor dietary habits (55%), frequent alcohol consumption (56%), and smoking (53%), with “do not know how to assess” (14–19%) and “delegated to other professionals” (7–21%) being the most common reasons. For frequent alcohol consumption and smoking, a certain proportion of physical therapists (11%) selected “not considered necessary.” In the metabolic factors, high non-assessment rates were observed for obesity (36%) and high blood glucose (59%), and the main reason was “delegated to other professionals” (13% and 25%, respectively). Notably, only four physical therapists (3%) reported assessing all items across the five domains.

### Intervention for LBP–related factors and reasons for non-intervention implementation

The number of physical therapists who did not implement each LBP-related intervention and the reasons for doing so are presented in Table 4. Differences in intervention implementation across factors are shown in Fig. 2. The number of physical therapists implementing interventions for biomechanical factors showed a pattern similar to that observed for assessments and was significantly higher than that for psychological, social, and metabolic factors (Holm-corrected *p* < 0.05, φ = 0.16–0.38).

**Table 4:** Reasons for not implementing interventions for each low back pain–related factor.

Domain	Factor not addressed	Overall (not intervened)	Not assessed	Do not know how to provide intervention	Lack of intervention skills	Lack of time	Patient discomfort with the intervention	Lack of necessary equipment	Not allowed by facility policy	No eligible patients	Not considered necessary	Delegated to other professionals
Biomechanical factors	Trunk muscle strength/endurance	17 (12)	2 (1)	4 (3)	11 (8)	0 (0)	1 (1)	0 (0)	0 (0)	0 (0)	0 (0)	0 (0)
	Overactivity of trunk muscles	34 (24)	6 (4)	18 (13)	16 (11)	0 (0)	0 (0)	0 (0)	0 (0)	0 (0)	0 (0)	0 (0)
	Flexibility of adjacent regions (hip, thoracic spine)	25 (18)	5 (4)	10 (7)	13 (9)	0 (0)	0 (0)	0 (0)	0 (0)	0 (0)	0 (0)	0 (0)
	Lumbar motor control	52 (37)	14 (10)	25 (18)	24 (17)	2 (1)	1 (1)	0 (0)	0 (0)	0 (0)	0 (0)	0 (0)
	Body perception	64 (46)	17 (12)	31 (22)	21 (15)	1 (1)	0 (0)	0 (0)	0 (0)	2 (1)	1 (1)	1 (1)
	Postural and movement dysfunction	17 (12)	2 (1)	5 (4)	10 (7)	1 (1)	1 (1)	0 (0)	0 (0)	0 (0)	0 (0)	0 (0)
Psychological factors	Anxiety or depressive symptoms	83 (59)	28 (20)	32 (23)	24 (17)	2 (1)	2 (1)	2 (1)	0 (0)	3 (2)	2 (1)	11 (8)
	Kinesiophobia	68 (49)	25 (18)	27 (19)	19 (14)	2 (1)	2 (1)	1 (1)	0 (0)	3 (2)	2 (1)	6 (4)
	Low self-efficacy	79 (56)	28 (20)	27 (19)	22 (16)	2 (1)	2 (1)	1 (1)	0 (0)	2 (1)	2 (1)	10 (7)
Social factors	Psychological stress (family or workplace)	73 (52)	22 (16)	23 (16)	13 (9)	2 (1)	2 (1)	1 (1)	0 (0)	5 (4)	4 (3)	16 (11)
	Work environment (desk work, heavy lifting)	32 (23)	5 (4)	11 (8)	9 (6)	2 (1)	0 (0)	2 (1)	0 (0)	4 (3)	1 (1)	3 (2)
Lifestyle factors	Sleep	73 (52)	22 (16)	29 (21)	13 (9)	1 (1)	0 (0)	1 (1)	0 (0)	2 (1)	3 (2)	16 (11)
	Physical activity	31 (22)	7 (5)	11 (8)	6 (4)	2 (1)	0 (0)	4 (3)	0 (0)	2 (1)	0 (0)	3 (2)
	Dietary habits	79 (56)	24 (17)	15 (11)	4 (3)	4 (3)	1 (1)	1 (1)	0 (0)	2 (1)	4 (3)	33 (24)
	Alcohol consumption	86 (61)	28 (20)	17 (12)	6 (4)	2 (1)	1 (1)	1 (1)	0 (0)	2 (1)	5 (4)	36 (26)
	Smoking	84 (60)	27 (19)	17 (12)	6 (4)	2 (1)	1 (1)	1 (1)	0 (0)	2 (1)	5 (4)	35 (25)
Metabolic factors	Obesity	62 (44)	16 (11)	14 (10)	7 (5)	4 (3)	1 (1)	1 (1)	0 (0)	3 (2)	1 (1)	24 (17)
	Hyperglycemia	83 (59)	23 (16)	14 (10)	6 (4)	5 (4)	2 (1)	1 (1)	0 (0)	2 (1)	3 (2)	38 (27)
All items treated		2 (1)	–	–	–	–	–	–	–	–	–	–

Values represent the number (percentage) of respondents who selected each reason for not providing interventions for each factor.

The percentages were calculated based on the total number of respondents (*n* = 140).

Multiple answers were allowed. Therefore, the sum of the reasons did not equal the overall number of respondents who did not provide interventions for each factor.

The psychological and metabolic factors also had significantly fewer physical therapists implementing interventions than the biomechanical, social (Holm-corrected *p* < 0.01, φ = 0.24), and lifestyle factors (Holm-corrected *p* < 0.01, φ = 0.26).

Among the biomechanical factors, high non-intervention rates were observed for overactivation or imbalance of trunk muscles (24%), impaired lumbar motor control (37%), and altered body perception (46%). The main reasons were “do not know how to provide intervention” (13–22%) and “lack of intervention skills” (11–17%). Among the psychological factors, all items showed high non-intervention rates, and the main reasons were “not assessed” (18–20%), “do not know how to provide intervention” (19–23%), and “lack of intervention skills” (14–17%). In addition, “delegated to other professionals” (6–11%) was also frequently reported. Among the social factors, stress (family or workplace) showed a high non-intervention rate (52%), mainly due to “not assessed” (16%) and “do not know how to provide intervention” (16%). For the lifestyle factors, high non-intervention rates were observed for poor sleep (quantity/quality) (52%), poor dietary habits (56%), frequent alcohol consumption (61%), and smoking (60%), with “not assessed” (16–20%) and “delegated to other professionals” (11–25%) being the most common reasons. In the metabolic factors, high non-intervention rates were observed for obesity (44%) and high blood glucose (59%), and the main reason was “delegated to other professionals” (17% and 27%, respectively). Only two physical therapists (1%) reported implementing interventions for all items across the five domains.

### Types of interventions used for patients with LBP

The types of interventions implemented for patients with LBP are presented in Table 5.

**Table 5:** Types of interventions implemented for patients with low back pain.

Type of intervention	*n* (%)
Exercise therapy for lower limbs or whole body (including strengthening and walking)	131 (94)
Trunk muscle strength and endurance training	132 (94)
Lumbar motor control training	82 (59)
Manual therapy for lumbar muscles/fascia (including massage, myofascial release)	112 (80)
Flexibility exercises for non-lumbar muscles/fascia (including stretching)	125 (89)
Spinal mobilization	71 (51)
Directional preference exercise (McKenzie method)	20 (14)
Posture and movement instruction (including posture correction, lifting techniques)	118 (84)
Advice on work environment	85 (61)
Nutritional guidance (including anti-inflammatory diet, vitamin D supplementation)	16 (11)
Lifestyle guidance (activity, sleep, smoking, alcohol)	67 (48)
Graded exposure therapy (progressive exposure to fear-avoidance behaviors)	5 (4)
Mindfulness-based stress reduction	5 (4)
Pain Neuroscience Education	14 (10)
Physical modalities (including heat, electrical stimulation, shockwave therapy)	70 (50)
Cognitive behavioral therapy (including psychologically informed interventions)	20 (14)
Acceptance and commitment therapy (including mindfulness)	0 (0)
Cognitive functional therapy (including movement re-education)	8 (6)
None	1 (1)

Values represent the number (percentage) of respondents who reported providing each intervention for patients with low back pain. Percentages were calculated based on the total number of respondents (*n* = 140). Multiple answers were allowed.

The most frequently implemented interventions were exercise therapy for the lower limbs or whole body (including strengthening and walking) (94%) and trunk muscle strength and endurance training (94%); followed by flexibility exercises for non-lumbar muscles/fascia (including stretching) (89%); posture and movement instruction (including posture correction and lifting techniques) (84%); and manual therapy for lumbar muscles/fascia (including massage and myofascial release) (80%).

In contrast, lumbar motor control training (59%) and spinal mobilization (51%) were implemented by approximately half of the physical therapists.

Psychological and behavioral approaches have been reported to be less frequent than physical interventions. These included pain neuroscience education (10%), cognitive-behavioral therapy-related or psychologically informed approaches (14%), and mindfulness-based interventions (4%) (Table 5).

### Factors and background characteristics associated with non-implementation

Based on the quantification theory type III results, the first dimension of the physical therapists’ latent characteristics (contribution rate: 32.4%) represented the type of facility. High scores indicated that physical therapists worked in orthopedic clinics, and low scores, that they worked in non-clinic facilities. Regarding patient characteristics, two dimensions were extracted based on the LBP symptoms that were primarily managed by the physical therapists (first-dimension contribution rate: 40.1%; second-dimension: 22.8%; cumulative contribution: 62.9%). The first dimension was the acute–chronic spectrum (high scores indicated chronic LBP), whereas the second dimension represented the presence or absence of lower-limb neurological symptoms (high scores indicated the presence of neurological symptoms). Based on these results, discriminant analysis was conducted using three independent variables: (1) working in an orthopedic clinic, (2) primarily managing patients with chronic LBP, and (3) primarily managing patients with lower-limb neurological symptoms (Table 6).

**Table 6:** Discriminant analysis of reasons for non-implementation of assessment/intervention and related background characteristics.

		Domain	Factor not addressed	Significance of Discriminant Function			Characteristics of Work Facility				Classification of Low Back Pain Symptoms							
							Working in a Clinic (+)				Presence of Lower Limb Neurological Symptoms (+)				Chronicity (+ indicates chronic)			
				Wilks’ λ	Chi-square Value	p-value	Unstandardized Discriminant Coefficient (Not Reported as a Reason)	Unstandardized Discriminant Coefficient (Reported as a Reason)	Standardized Discriminant Coefficient	p-value	Unstandardized Discriminant Coefficient (Not Reported as a Reason)	Unstandardized Discriminant Coefficient (Reported as a Reason)	Standardized Discriminant Coefficient	p-value	Unstandardized Discriminant Coefficient (Not Reported as a Reason)	Unstandardized Discriminant Coefficient (Reported as a Reason)	Standardized Discriminant Coefficient	p-value
Assessments	Do not know how to assess	Biomechanical factors	Impaired lumbar motor control	0.94	9.12	0.03	0.18	-0.13	0.14	0.10	-0.06	0.45	0.22	0.01	-0.12	-0.34	0.10	0.25
		Psychological factors	Anxiety or depressive symptoms	0.93	9.64	0.02	0.14	0.12	0.01	0.95	-0.02	0.25	0.12	0.16	-0.07	-0.62	0.24	0.00
		Lifestyle factors	Poor dietary habits	0.91	12.29	0.01	0.05	0.60	0.25	0.00	0.08	-0.29	0.16	0.05	-0.12	-0.34	0.10	0.24
			Alcohol consumption	0.94	8.02	0.05	0.09	0.43	0.15	0.07	0.06	-0.23	0.13	0.13	-0.10	-0.44	0.15	0.08
			Smoking	0.92	10.67	0.01	0.08	0.53	0.19	0.03	0.06	-0.28	0.13	0.11	-0.10	-0.52	0.17	0.04
		Metabolic factors	High blood glucose	0.91	12.93	0.00	0.07	0.71	0.26	0.00	0.05	-0.24	0.11	0.18	-0.11	-0.49	0.15	0.08
	Lack of assessment skills	Biomechanical factors	Impaired lumbar motor control	0.91	12.68	0.01	0.20	-0.14	0.16	0.06	-0.10	0.50	0.28	0.00	-0.13	-0.24	0.05	0.53
	Delegated to other professionals	Metabolic factors	High blood glucose	0.94	8.13	0.04	0.17	0.04	0.07	0.43	-0.10	0.37	0.24	0.00	-0.15	-0.17	0.01	0.86
Interventions	Do not know how to provide intervention	Biomechanical factors	Overactivation or imbalance of trunk muscles	0.94	9.00	0.03	0.18	-0.18	0.15	0.08	-0.04	0.42	0.18	0.03	-0.11	-0.46	0.14	0.10
			Impaired lumbar motor control	0.91	12.31	0.01	0.20	-0.15	0.16	0.05	-0.08	0.50	0.26	0.00	-0.11	-0.32	0.09	0.26

The results of the discriminant analysis showed that primarily managing patients with lower limb neurological symptoms was a significant discriminant factor for “do not know how to assess impaired lumbar motor control” (*p* = 0.01, standardized discriminant coefficient = 0.22) and “lack of assessment skills for impaired lumbar motor control” (*p* < 0.01, standardized discriminant coefficient = 0.28). It was also a significant discriminant factor for “delegated to other professionals” in assessing high blood glucose (*p* < 0.01, standardized discriminant coefficient = 0.24) and “do not know how to provide intervention” for overactivation or imbalance of trunk muscles (*p* = 0.03, standardized discriminant coefficient = 0.18) and impaired lumbar motor control (*p* < 0.01, standardized discriminant coefficient = 0.26). In addition, primarily managing patients with acute LBP was a significant discriminant factor for “do not know how to assess anxiety or depressive symptoms” (*p* < 0.01, standardized discriminant coefficient = 0.24) and “do not know how to assess smoking” (*p* = 0.04, standardized discriminant coefficient = 0.17). Furthermore, working in an orthopedic clinic was identified as a discriminant factor for “do not know how to assess poor dietary habits” (*p* < 0.01, standardized discriminant coefficient = 0.25), smoking (*p* = 0.03, standardized discriminant coefficient = 0.19), and high blood glucose (*p* < 0.01, standardized discriminant coefficient = 0.26).

## Discussion

This study clarified the implementation status of multifaceted assessments and interventions for LBP among physical therapists, and comprehensively examined the reasons for non-implementation and related background characteristics. These findings suggest that the management of LBP by physical therapists tends to focus on biomechanical factors, whereas assessments and interventions targeting psychological, lifestyle, and metabolic factors are less frequently integrated into physical therapy practice. Furthermore, structural barriers, including insufficient knowledge and skills, time constraints, and role boundaries with other professionals were identified as underlying factors for non-implementation. These barriers varied according to clinical contexts, including facility type and patient characteristics. The findings are discussed further below.

This study revealed that, compared with biomechanical factors, including decreased flexibility of adjacent regions (hip and thoracic spine) and lifestyle-related factors, the number of physical therapists who implemented assessments and interventions addressing psychological and metabolic factors was significantly lower. In particular, the number of physical therapists implementing assessments or interventions for anxiety or depressive symptoms, kinesiophobia (fear of movement), low self-efficacy, poor sleep (quantity/quality), poor dietary habits, smoking, and frequent alcohol consumption was less than half. These findings indicate a potential gap between the available evidence on comprehensive LBP management and its application in physical therapy practice. The observed reliance on delegation to other professionals may reflect appropriate clinical decision-making within multidisciplinary care rather than a limitation of physical therapy practice. Regarding specific intervention strategies, biomechanical approaches, including trunk muscle strength and endurance training, exercise therapy for the lower limbs or whole body, posture and movement instructions, and manual therapy for the lumbar muscles/fascia, were frequently implemented.

In contrast, psychologically or behaviorally informed interventions, including approaches such as patient education, communication strategies, psychologically informed care, and specialized interventions (e.g., cognitive behavioral therapy, acceptance and commitment therapy, graded exposure therapy, and mindfulness-based stress reduction), were less frequently reported. This trend is consistent with previous studies indicating that many healthcare providers maintain a predominantly biomedical belief system^41,42^, which may influence patients’ beliefs and behaviors related to LBP^43^. Excessive emphasis on biomedical approaches has also been associated with restricted physical activity, reduced work participation, and an increased use of medical interventions^30,41^. Considering the professional scope of physical therapists, it is natural that assessments and interventions should focus primarily on biomechanical factors. However, the findings of this study suggest that it may be important to consider how psychological, lifestyle, and metabolic factors can be incorporated into physical therapy practice, not only through direct management, but also through interprofessional collaboration. These findings may help clarify how such factors can be addressed in physical therapy practice and contribute to defining role delineation and clinical decision-making frameworks in multidisciplinary care.

Across many items, the main reasons for not conducting assessments or interventions were “Do not know how to assess or provide intervention” and “Lack of assessment or intervention skills”. This tendency was particularly evident for psychological factors. While psychosocial approaches have been shown to be effective^34,35,44^, their clinical implementation remains limited because of factors such as lack of time, insufficient educational opportunities, limited clinical confidence, and challenges in integrating psychological and biomedical approaches. These findings indicate that insufficient training within educational curricula remains a major barrier. Similarly, in the present study, psychological factors frequently showed association with knowledge and skill deficits as well as time constraints, reflecting a consistent trend with previous reports.

Furthermore, differences in implementation were also observed among the biomechanical factors. Compared with decreased trunk muscle strength/endurance, postural/movement dysfunction, and decreased flexibility of adjacent regions, fewer physical therapists implemented assessments or interventions for overactivation or imbalance of trunk muscles, impaired lumbar motor control, and altered body perception. The assessment and intervention methods for these biomechanical factors vary depending on the focus (e.g., sensory, muscular, or joint) and standardized procedures are lacking^45,46^. In addition, the complexity of lumbar motor control and its interaction with psychosocial factors may further hinder clinical implementation^46–48^. Therefore, many physical therapists may perceive a lack of knowledge and technical skills regarding these factors and may then refrain from performing related assessments or interventions. Establishing standardized and clinically feasible approaches for assessments and interventions on sensorimotor control is thus a key challenge against improving implementation in clinical practice.

Another notable reason for the non-implementation of lifestyle and metabolic factors was the high frequency of “delegation to other professionals.” This tendency may reflect long-standing biomedical traditions and societal expectations as well as uncertainties regarding the scope of practice and hesitancy to assume roles typically attributed to other professionals. The lack of clarity in defining professional roles and boundaries has been identified as a major barrier to implementing a biopsychosocial model^49^. However, the delegation to other professionals should not necessarily be considered negative. When adequate interprofessional collaboration is established with clearly defined roles and shared responsibilities, continuity and quality of care can be maintained even without direct intervention by physical therapists. In this context, physical therapists may play a role in appropriately referring patients who require care under these domains to other professionals, as well as integrating elements of psychological, social, lifestyle, and metabolic considerations into biomechanical interventions (e.g., adapting communication strategies and supporting a shift in focus from pain to activity). These findings highlight the importance of such approaches in physical therapy practice. Conversely, when such delegation is only nominal and lacks substantive information sharing or well-defined collaborative structures, there is a risk that interventions targeting specific factors may be overlooked. Therefore, clarifying and strengthening the mutual understanding of professional responsibilities among physical therapists and other healthcare providers, as well as fostering an environment that supports inter-professional collaboration, may be important^44,49^. However, it remains uncertain whether the responses indicating “delegation to other professionals” in this study reflect true collaborative practice or merely a perceived division of roles. Further investigation is needed to clarify the nature of this delegation and its implications in clinical practice.

Discriminant analysis revealed that facility type (working in an orthopedic clinic) and patient characteristics (chronic LBP and presence of lower limb neurological symptoms) were significant discriminant factors for reasons for non-implementation. Specifically, among physical therapists who responded with “do not know how to assess” or “lack of assessment skills for impaired lumbar motor control,” a significant discriminant factor was identified as primarily managing patients with lower limb neurological symptoms. Based on the unstandardized discriminant coefficients, physical therapists who frequently treated patients with neurological symptoms tended to develop greater deficits in knowledge and skills related to the assessment and intervention of motor control. Patients with LBP accompanied by neurological symptoms often present with multifaceted problems, including lower-limb weakness, pain, sensory disturbances, and associated activity limitations.

Thus, clinical priorities are often directed toward improving activities of daily living and overall functional mobility, whereas higher-level tasks, including those related to sensorimotor control (including lumbar motor control), are likely to receive less attention. Moreover, neurological symptoms often involve structural pathologies, including intervertebral disc degeneration or spinal canal stenosis^50^, in which immediate improvement is difficult to achieve with motor control training alone. These clinical characteristics may contribute to the perception of inadequate knowledge and technical skills regarding the assessment of motor control. Therefore, developing simple, reliable, and reproducible assessment methods for lumbar motor control that are applicable even to patients with neurological symptoms and educational dissemination of their clinical relevance is warranted.

In addition, among physical therapists who indicated that they did not know how to assess anxiety or depressive symptoms, a significant discriminating factor was identified as primarily managing patients with acute LBP. In the acute phase, biomechanical factors, including local inflammation and range-of-motion limitation, tend to have a greater impact; thus, assessment and intervention of psychological factors assume a lower clinical priority^51^. This finding reflects the clinical tendency to prioritize physical recovery in the early stages of LBP, while underrecognizing the role of psychological contributors. By contrast, chronic LBP is more strongly influenced by psychological factors, and the effectiveness of multimodal interventions incorporating psychological components has been reported^26,52^. However, when psychological factors are insufficiently assessed during the acute phase, the risk of pain and activity limitation becoming chronic increases^53,54^. Taken together, these findings suggest that screening for psychological and behavioral factors may be considered an important component of the initial evaluation to prevent the transition from acute to chronic LBP, not as an “additional assessment specific to the chronic phase.”

Furthermore, among lifestyle factors, including poor dietary habits, smoking, and frequent alcohol consumption, working in an orthopedic clinic was identified as a significant discriminant factor for the response, “Do not know how to assess.” According to the unstandardized discriminant coefficients, physical therapists working in orthopedic clinics were more likely to report uncertainty regarding the assessment methods of lifestyle-related items. Compared to inpatient or long-term care facilities, orthopedic clinics often provide fewer opportunities to obtain information about patients’ daily living from other professionals, including nurses or dietitians, and time constraints may also limit physical therapists’ direct involvement in lifestyle guidance. Such structural conditions may be one of the factors explaining why working in a clinic has emerged as a discriminant variable associated with the non-implementation of lifestyle and metabolic factors.

This study’s findings provide several important insights for future educational and practical strategies aimed at implementing multimodal assessments and interventions for LBP in physical therapy practice. First, the results highlighted that establishing an educational framework may be key to facilitating clinical implementation. Many physical therapists reported “Do not know how to assess/intervene” or “Lack of assessment/intervention skills,” particularly for sensorimotor, psychological, lifestyle, and metabolic factors. This indicates gaps in knowledge and technical proficiency, which likely reflects insufficient educational opportunities and limited curricular emphasis on these factors. Therefore, undergraduate and postgraduate education should include foundational frameworks for psychologically and behaviorally informed interventions, such as pain neuroscience education and behavioral support related to sleep, physical activity, and nutrition, as well as standardized methods for sensorimotor control assessment.

Second, it is necessary to redefine physical therapists’ professional roles in addressing lifestyle and metabolic factors and to clarify their scope of practice. In this study, a relatively high proportion of physical therapists selected “Delegated to other professionals” or “Not considered necessary” for dietary habits, smoking, and blood glucose, suggesting a tendency among physical therapists to position these factors outside their own professional domain. However, lifestyle and metabolic factors are closely associated with LBP chronicity and recurrence^18–20^ and represent modifiable factors that are strongly linked to physical activity and exercise therapy. Therefore, physical therapists may play a role in primary-level interventions including promoting physical activity, providing sleep hygiene education, and motivating dietary improvement. This should be undertaken in collaboration with other healthcare professionals, including physicians, registered dietitians, and psychologists, within a “primary intervention plus professional collaboration” model as needed. The finding that a high proportion of physical therapists reported “Delegated to other professionals” suggests the existence of role division among healthcare professionals. However, it remains unclear whether such delegation is based on effective interprofessional collaboration with adequate information sharing and shared goals. Hellman et al.^49^ reported that in team-based rehabilitation for patients with LBP, clear interprofessional collaboration and goal alignment will result in improved patient outcomes. Taken together, these findings indicate that achieving comprehensive management, including that of lifestyle and metabolic factors, requires clarification and reconstruction of a collaborative framework in which multiple professionals, including physical therapists, share roles and responsibilities.

Third, the finding that many physical therapists reported time constraints in assessing or addressing psychological factors indicates the need to improve clinical efficiency.

Introducing brief but reliable screening tools, including the STarT Back Tool or the Örebro Musculoskeletal Pain Screening Questionnaire^52^, could standardize the “Screen → Stratify → Treat” process and facilitate the integration of multimodal factors into routine clinical practice. Furthermore, differences in the background characteristics identified in this study could be utilized to design customized educational and training programs tailored to specific work settings and patient populations.

Thus, this study clarified the key challenges and directions for implementing multimodal assessments and interventions for LBP by physical therapists, including educational, collaborative, and system-level factors. By identifying the factors that are less frequently addressed and their underlying reasons, these findings provide a foundation for developing practical evidence-based implementation strategies in clinical settings, which may contribute to improving patient outcomes and reducing the burden of LBP.

This study had several limitations. First, the survey was limited to Ishikawa Prefecture in Japan, and regional differences in culture and healthcare systems may restrict the generalizability of our findings. Previous studies have reported that the beliefs, clinical orientations, and guideline recommendations of physical therapists regarding LBP vary across regions and educational backgrounds^55^. In addition, differences in healthcare systems and practice settings, such as the predominance of hospital-based versus outpatient-based care, may contribute to such variations and further limit the generalizability of our findings. Future studies should include multiple regions, educational systems, and practical settings in order to verify these findings.

Second, due to the self-reported nature of the questionnaire, it is difficult to completely eliminate social desirability bias (i.e., the tendency to report “implementation” behaviors). Conversely, the participants may have underestimated their actual practices because of their awareness of the research team’s expertise and reputation. Furthermore, the physical therapists who responded to this survey were likely more motivated and research- or education-oriented (self-selection bias), which could have resulted in an overestimation of implementation rates relative to the broader clinical population.

Third, although LBP-related factors were classified into five domains for analysis, the potential interactions among the domains could not be sufficiently examined. For example, kinesiophobia can be considered as a psychological factor, yet it may also influence physical activity levels and metabolic status through activity avoidance^56^. However, these chain mechanisms were beyond the scope of this study.

Fourth, although the questionnaire included an open-ended section, the response structure largely utilized predefined choices determined by the research team. Consequently, certain respondent or patient characteristics (including issues related to LBP) and potential barriers not anticipated during the design phase may not have been fully captured.

Fifth, the questionnaire used in this study was developed as a pragmatic, study-specific instrument and did not undergo full-formal scale development or psychometric validation. Although content validity and comprehensibility were assessed through expert review and cognitive pretesting, more rigorous procedures such as quantitative content validity indices, large-scale pilot testing, and construct validation were not performed. Therefore, the questionnaire’s measurement properties should be interpreted with caution.

Finally, because of the sample size requirement for discriminant analysis (*n* ≥ 5 × k), some items were excluded from the analysis, which may have limited the ability to clarify the overall structure of relationships. Future research should employ larger and more stratified samples to confirm and expand upon these findings.

## Conclusions

This study comprehensively examined multimodal assessments and intervention implementation status for LBP by physical therapists as well as the reasons for non-implementation and contextual background characteristics. These findings suggest that the physical therapy practice in this study tended to focus more on biomechanical factors, whereas psychological and metabolic factors were addressed less frequently. Underlying these gaps are structural barriers beyond individual factors, including limited educational opportunities, time constraints, and unclear professional boundaries between disciplines. Moreover, the reasons for non-implementation differed depending on the clinical context, including the work setting and patient characteristics. These findings highlight the importance of comprehensive strategies encompassing education, interprofessional collaboration, and system-level approaches to promote the clinical implementation of multimodal LBP management by physical therapists.

## Electronic Supplementary Material

Below is the link to the electronic supplementary material.

Supplementary Material 1

Supplementary Material 2

## Data Availability

The data associated with the present study are not publicly available but are available from the corresponding author upon reasonable request.
