# Supplementary material for: Implementation status and barriers to multimodal management of low back pain among physical therapists: A cross-sectional study
Source: Sci Rep. 2026 Apr 24;16:19083. doi: 10.1038/s41598-026-50076-2 (PMC13280009; doi:10.1038/s41598-026-50076-2)
Supplement: Supplementary file 1 — Supplementary Material 1 [file 41598_2026_50076_MOESM1_ESM.docx]

**Questionnaire items and response options**

| **Characteristics** | | **Options** | **Question type** |
| --- | --- | --- | --- |
| Sex | | Male / Female | Single choice |
| Age of respondents | | 20–29 years 30–39 years 40–49 years  50–59 years 60 years and above | Single choice |
| Clinical experience | |  | Free text |
| Work setting | | Acute care hospital Rehabilitation hospital Orthopedic clinic Home-visit rehabilitation service Nursing facility Sports team Others | Multiple choice |
| Frequency of treating patients with low back pain | | Almost every day Several times a week Several times a month Several times in 6 months Several times a year Rarely treat | Single choice |
| Age group of patients with low back pain | | Under 10 years 10–19 years 20–29 years 30–39 years 40–49 years 50–59 years 60–69 years 70–79 years 80 years and above Others | Multiple choice |
| Clinical stage of low back pain and leg symptoms | | Acute LBP without leg symptoms Chronic LBP without leg symptoms Acute LBP with leg symptoms Chronic LBP with leg symptoms Others | Multiple choice |
| Patient condition mainly managed | | Postoperative (inpatient) Postoperative (outpatient) Non-surgical (conservative) management (inpatient) Non-surgical (conservative) management (outpatient) Others | Multiple choice |
| Awareness of low back pain–related factors | | Decreased trunk muscle strength/endurance Overactivation or imbalance of trunk muscles Decreased flexibility of adjacent regions (hip, thoracic spine)  Impaired lumbar motor control Altered body perception Postural or movement dysfunction Anxiety or depressive symptoms Kinesiophobia (fear of movement) Low self-efficacy Psychological stress (family or workplace) Work environment (including prolonged desk work, heavy lifting) Poor sleep (quantity/quality) Low physical activity (lack of exercise habits) Poor dietary habits Frequent alcohol consumption Smoking Obesity High blood glucose | Multiple choice |
| Factors considered to be associated with low back pain that are not assessed, and the corresponding reasons | Decreased trunk muscle strength/endurance | Do not know how to assess Lack of assessment skills Lack of time Patient discomfort with the assessment Lack of necessary equipment Not allowed by facility policy No eligible patients Not considered necessary Delegated to other professionals Others | Multiple choice |
|  | Overactivation or imbalance of trunk muscles |  |  |
|  | Decreased flexibility of adjacent regions (hip, thoracic spine) |  |  |
|  | Impaired lumbar motor control |  |  |
|  | Altered body perception |  |  |
|  | Postural/movement dysfunction |  |  |
|  | Anxiety or depressive symptoms |  |  |
|  | Kinesiophobia (fear of movement) |  |  |
|  | Low self-efficacy |  |  |
|  | Psychological stress (family or workplace) |  |  |
|  | Work environment (including prolonged desk work, heavy lifting) |  |  |
|  | Poor sleep (quantity/quality) |  |  |
|  | Low physical activity (lack of exercise habits) |  |  |
|  | Poor dietary habits |  |  |
|  | Frequent alcohol consumption |  |  |
|  | Smoking |  |  |
|  | Obesity |  |  |
|  | High blood glucose |  |  |
|  | All items evaluated |  |  |
| Factors considered to be associated with low back pain that are not addressed through intervention, and the corresponding reasons | Decreased trunk muscle strength/endurance | Not assessed Do not know how to provide intervention Lack of intervention skills Lack of time Patient discomfort with the intervention Lack of necessary equipment Not allowed by facility policy No eligible patients Not considered necessary Delegated to other professionals Others | Multiple choice |
|  | Overactivation or imbalance of trunk muscles |  |  |
|  | Decreased flexibility of adjacent regions (hip, thoracic spine) |  |  |
|  | Impaired lumbar motor control |  |  |
|  | Altered body perception |  |  |
|  | Postural/movement dysfunction |  |  |
|  | Anxiety or depressive symptoms |  |  |
|  | Kinesiophobia (fear of movement) |  |  |
|  | Low self-efficacy |  |  |
|  | Psychological stress (family or workplace) |  |  |
|  | Work environment (including prolonged desk work, heavy lifting) |  |  |
|  | Poor sleep (quantity/quality) |  |  |
|  | Low physical activity (lack of exercise habits) |  |  |
|  | Poor dietary habits |  |  |
|  | Frequent alcohol consumption |  |  |
|  | Smoking |  |  |
|  | Obesity |  |  |
|  | High blood glucose |  |  |
|  | All items treated |  |  |
| Interventions implemented for patients with low back pain | | Exercise therapy for lower limbs or whole body (including strengthening and walking) Trunk muscle strength and endurance training Lumbar motor control training Manual therapy for lumbar muscles/fascia (including massage, myofascial release) Flexibility exercises for non-lumbar muscles/fascia (including stretching)  Spinal mobilization Directional preference exercise (McKenzie method) Posture and movement instruction (including posture correction, lifting techniques) Advice on work environment Nutritional guidance (including anti-inflammatory diet, vitamin D supplementation) Lifestyle guidance (activity, sleep, smoking, alcohol) Graded exposure therapy (progressive exposure to fear-avoidance behaviors) Mindfulness-based stress reduction Pain Neuroscience Education Physical modalities (including heat, electrical stimulation, shockwave therapy) Cognitive behavioral therapy (including psychologically informed interventions) Acceptance and commitment therapy (including mindfulness) Cognitive functional therapy (including movement re-education) None | Multiple choice |
